# Opportunities of connectomic neuromodulation

**DOI:** 10.1016/j.neuroimage.2020.117180

**Published:** 2020-07-20

**Authors:** Andreas Horn, Michael D. Fox

**Affiliations:** a Neurology Department, Movement Disorders and Neuromodulation Sectio Charité – University Medicine Berlin,, Charitéplatz 1, D-10117 Berlin, Germany; b Berenson–Allen Center for Non-invasive Brain Stimulation, Department of Neurology, Harvard Medical School and Beth Israel Deaconess Medical Center, United States; c Martinos Center for Biomedical Imaging, Departments of Neurology and Radiology, Harvard Medical School and Massachusetts General Hospital, United States; d Center for Brain Circuit Therapeutics, Departments of Neurology, Psychiatry, and Radiology, Harvard Medical School and Brigham and Women's Hospital, United States

**Keywords:** Deep brain stimulation, Connectomics, Functional MRI, fMRI, dMRI, Diffusion MRI, Tractography, Brain networks, Network fingerprinting, Brain stimulation, Neuromodulation

## Abstract

The process of altering neural activity – neuromodulation – has long been used to treat patients with brain disorders and answer scientific questions. Deep brain stimulation in particular has provided clinical benefit to over 150,000 patients. However, our understanding of how neuromodulation impacts the brain is evolving. Instead of focusing on the local impact at the stimulation site itself, we are considering the remote impact on brainregions connected to the stimulation site. Brain connectivity information derived from advanced magnetic resonance imaging data can be used to identify these connections and better understand clinical and behavioral effectsof neuromodulation. In this article, we review studies combining neuromodulation and brain connectomics, highlighting opportunities where this approach may prove particularly valuable. We focus on deep brain stimulation, but show that the same principles can be applied to other forms of neuromodulation, such as transcranial magnetic stimulation and MRI-guided focused ultrasound. We outline future perspectives and provide testable hypotheses for future work.

## 1. Introduction

The goal of this review is to provide an overview of the growing intersection between two fields: neuromodulation and brain connectomics. We highlight opportunities where this intersection may be leveraged to advance research and clinical care.

**Fig. 1.**
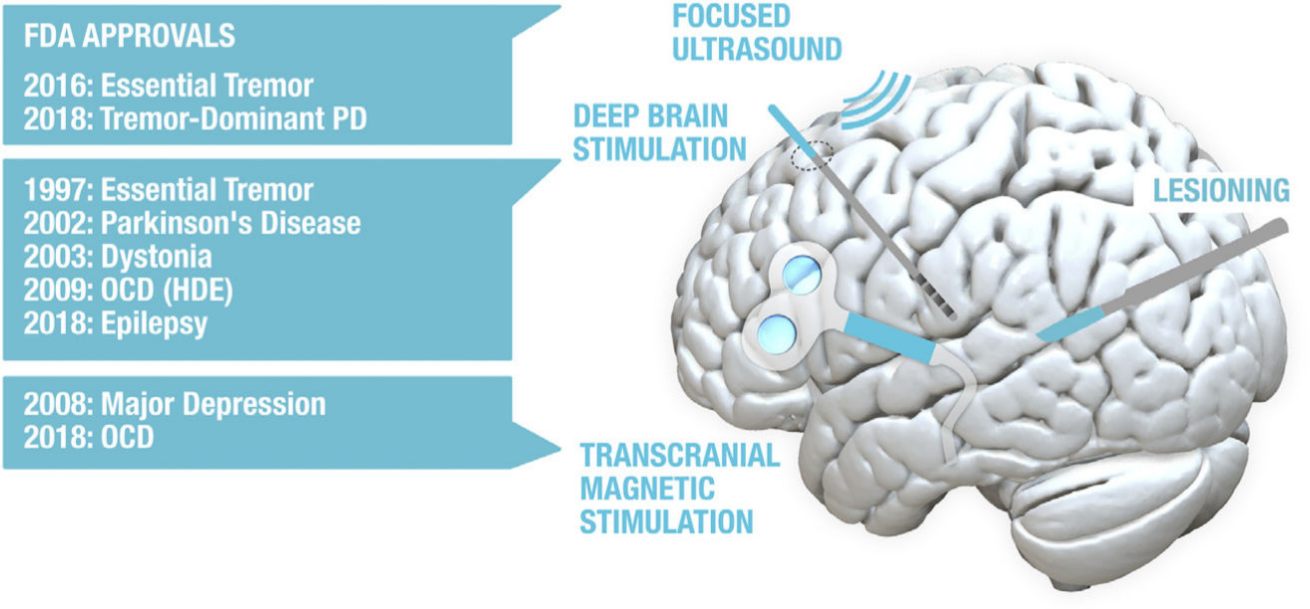
Methods used for clinical neuromodulation of the brain. List on the left shows recent device approvals issued by the U.S. Food and Drug Administration (FDA). HDE = Humanitarian device excemption. Various lesioning devices have been previously approved by the FDA for ablation of neural tissue (radiofrequency thermoablation, laser interstitial thermal therapy, sterotactic radiosurgery) with applications including thalamotomy for tremor, pallidotomy for Parkinson’s or dystonia, and cingulotomy for pain. Other technologies exist but have not been FDA approved for clinical neuromodulation of the brain (e.g. transcranial electrical current stimulation).

For the purpose of this review, we define neuromodulation as the process of altering neural activity using lesions, devices or electromagnetic energy to change human brain function. Neuromodulation includes *invasive* methods such as stereotactic lesions and deep brain stimulation (DBS) and *noninvasive* methods such as transcranial magnetic stimulation (TMS, Fig. 1).Neuromodulation can be reversible, as is the case with TMS or DBS, or irreversible, as is the case with brain lesions induced by neurosurgical ablation or MRI guided focused ultrasound (MRgFUS). Finally, neuromodulation can be used as a clinical treatment, to improve patient’s symptoms, or for scientific *research*, to better understand brain function.

In this review, we will touch on all forms of neuromodulation, but will focus on DBS as it is one of the most widely used clinical neuromodulation technologies with well-established therapeutic benefits. DBS leads to significant improvements of motor symptoms and quality of life inpatients with Parkinson’s Disease, Dystonia and Essential tial Tremor(Deuschl et al., 2006; Kupsch etal., 2006; Vitek et al., 2020).DBS is also FDA approved for the treatment of medication-refractory epilepsy (Salanova et al., 2015) and obsessive compulsive disorder (OCD, via humanitarian device exemption; (Anderson and Ahmed, 2003; Baldermann et al., 2019b; Franzini et al., 2010; Nuttin et al.,2003)). Finally, DBS has shown some promise in Tourette’s Syndrome (Ackermans et al., 2011), Huntington’s Disease (Gruber et al., 2014), Major Depression (Mayberg et al., 2005), alcohol addiction (Müller et al., 2009), and other emerging indications (Fox et al.,2014; Lozano et al., 2019).

The idea that brain connectivity may be important for understanding DBS and neuromodulation more generally is an old concept. Neurosurgical lesioning was performed as early as 1890, often with the goalof disrupting information flow between connected brain regions or brain *networks* (Gabriel and Nashold, 1998). For instance, Talairach and Leksell began lesioning the anterior limb of the internal capsule in patients with psychiatric disease with the goal of disrupting limbic input tothe prefrontal cortex (Feldman and Goodrich, 2001). Knight began lesioning white matter tracts below the caudate (subcaudate tractotomy) to disrupt the connection between orbitofrontal and limbic regions (W.S. Anderson, 2019).Some of the earliest studies of DBS adopted this same motivation, seeking to modulate the network of brain regions connected to the stimulation site (Montgomery and Gale, 2008).

**Fig. 2.**
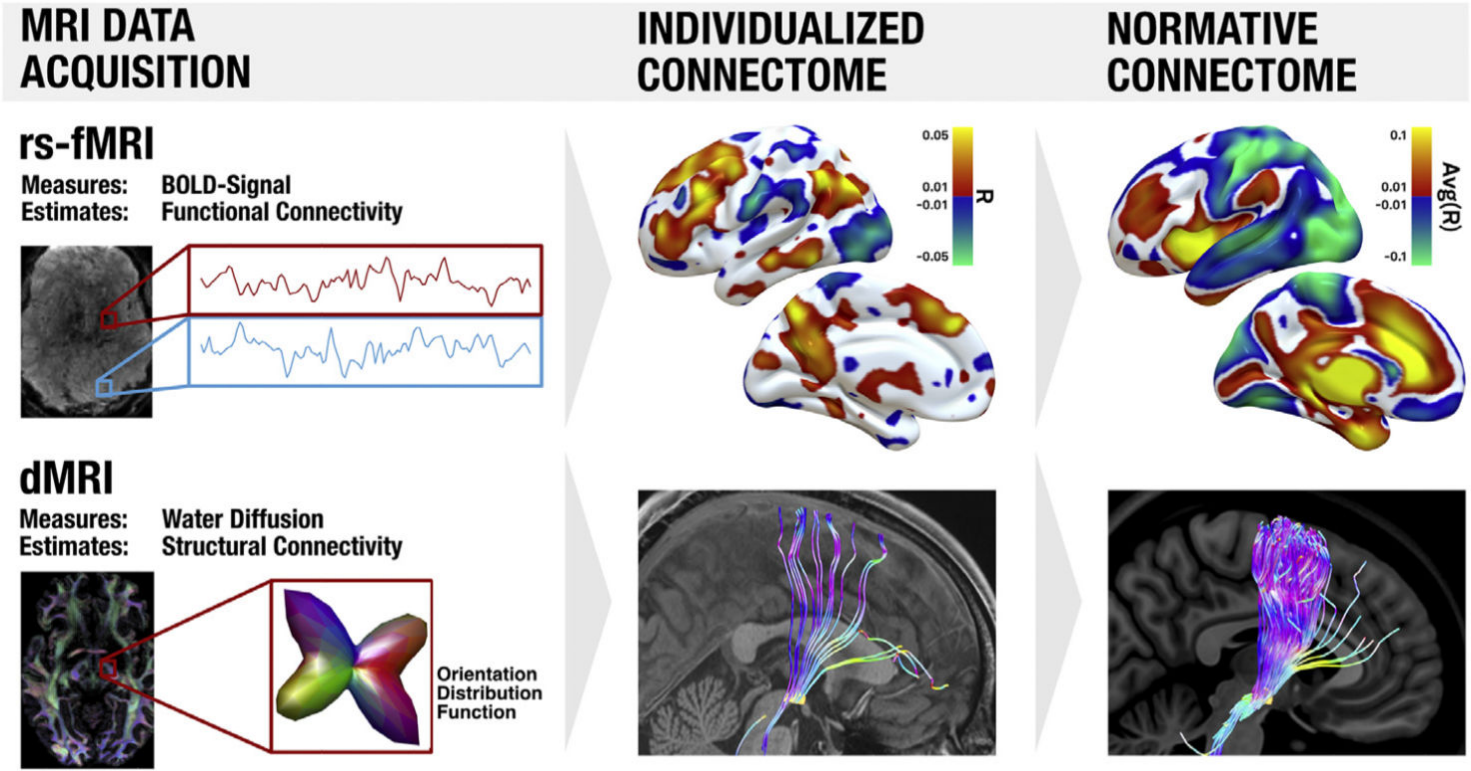
Noninvasive MRI based methods to estimate brain connectivity. Top: resting-state functional connectivity MRI (rs-fMRI) is based on spontaneous fluctuations in brain activity as indexed by the blood-oxygen-level-dependent (BOLD) signal. This signal is recorded from all voxels simultaneously, and voxels in which the fluctuations are correlated are considered functionally connected. Areas positively correlated to a seed region (right subthalamic nucleus, red box) are shown in hot colors, while regions negatively correlated (anticorrelated) to the seed region are shown in cool colors. Results based on a single subject are shown in the middle column (individualized connectome) while results based on 1000 subjects are shown in the right column (normative connectome). Bottom: diffusion-weighted imaging (dMRI) measures water diffusion which is anisotropic in the brain. In general, diffusion is stronger along the direction of larger fiber bundles as opposed to orthogonal to them. Based on local diffusion properties of each voxel (which can be represented as orientation distribution functions), tractography algorithms can estimate the location of white-matter bundles to provide an estimate of structural connectivity. White matter bundles passing through the subthalamic nucleusare shown for a single subject in the middle column and for a group of 1000 subjects in the right column. Displayed data are from the human connectome andgenome superstruct projects (Holmes et al., 2015; van Essen and Ugurbil, 2012).

Thus, the concept of using neuromodulation to target distributed brain *networks* is not new. What is new is our ability to visualize these networks in unprecedented detail and determine which networks are responsible for which symptoms. This ability was aided by two advancesin MRI technology: diffusion-weighted imaging based tractography (dMRI; to map structural connections, often expressed as streamline counts or average fractional anisotropy values (Jeurissen etal., 2019)) and resting-state functional magnetic resonance imaging (rs-fMRI; to map functional connections, of ten expressed as correlations between time series (Fox and Raichle, 2007a); Fig. 2). For the purpose of the present review, we will use the terms “structural connectivity” and “functional connectivity ”as a short-hand for describing findings derived from these techniques, respectively. When applying these measures to map connectivity between each region in the brain, a blueprint or wiring-diagram of the brain emerges, which we call the human connectome. The term *connectome* was coined by Olaf Sporns and Patric Hagmann in 2005 in close allegory to the human genome (Hagmann, 2005; Sporns et al., 2005). Their idea was to map the regions of the brain and their interconnections and formally describe their relationships using defined mathematical concepts. Large-scale academic efforts, such as the Human Connectome Project were soon launched with the goal of collecting high-quality brain connectivity data across a large number of subjects using specialized MR hardware (Van Essen et al., 2012). In recent years, wiring diagrams of the average human brain have emerged in form of *normative connectomes* (Holmes et al., 2015; Marek et al., 2011; Nooner et al., 2012; Van Essen et al., 2012; Yeo et al., 2011). These normative connectomes are robust, publicly available, and have proven useful for a range of clinical and scientific applications, including neuromodulation (Fox et al., 2014; Setsompop et al., 2013; Yeo et al., 2011). As such, much of this review focuses on the use of these normative connectomes. However, these same connectivity imaging techniques can also be used to construct wiring diagrams for individuals, which we refer to as *individualized connectomes*. Constructing a robust individualized connectome is currently difficult, requiring specialized expertise and long MRI scanning sessions that may be hard for patients to tolerate (Gordon et al., 2017; Jakab et al., 2016; Poldrack et al., 2015). However, as technology improves, individualized connectomes may complete ment or replace normative connectomes for understanding and guiding neuromodulation.

The value of the human connectome for guiding neuromodulation was recognized early on and referred to as “connectomic surgery” (Henderson, 2012). DBS was thought to work in part through modulation of remote brain regions connected to the site of stimulation (Montgomery and Gale, 2008). These remote effects of DBS on brain networks have been measured using a variety of techniques, including positron emission tomography, fMRI, magnetic encephalography, electroencephalography (EEG), local field potential recordings, and electrocorticography (Asanuma et al., 2006; Hirschmannetal., 2013; Neumann et al., 2015; Oswalet al.,2016). The human connectome promised to help us understand where these remote effects were coming from and even predict the remote effects based on connectivity with the stimulation site. Now that we had *mapped* the connectome, we were poisedto *apply* the connectome to address clinical questions and improve clinical treatment.

## 2. We have the connectome, (how) do we use it?

One example of how the connectome has been used to address clinical questions is in mapping symptoms caused by focal brain damage(see Fox (2018) for a review). Brain lesions in different patients that leadto the same clinical symptom are often scattered across the brain. In such cases, the symptom fails to map to a single brain region. By usingthe normative connectome, we can test whether these heterogenous lesion locations map to a single connected brain network (Fox,2018). For instance, lesion locations that cause amnesia occur in multiple different brain regions, but map to a single brain network defined by connectivity to the subiculum (Ferguson et al., 2019). Lesion locations associated with depression map to a brain network defined by connectivity tothe left dorsolateral prefrontal cortex (Padmanabhan et al., 2019). Lesion locations that result in tremor relief map to a single brain network defined by connectivity to the ventral intermediate nucleus of the thalamus (VIM) (Joutsa et al., 2018b). This same lesion network mapping approach has been applied to numerous other neurological and psychiatric symptoms (Fox, 2018). These studies used the normative connectome as an approximation of each patient’s individualized connectome at the time of the brain lesion. Despite methodological limitations (Cohen and Fox, 2020; Fox, 2018), the normative connectome has proven very useful in linking lesions in different locations causing the same symptom to a common neuroanatomic substrate.

**Fig. 3.**
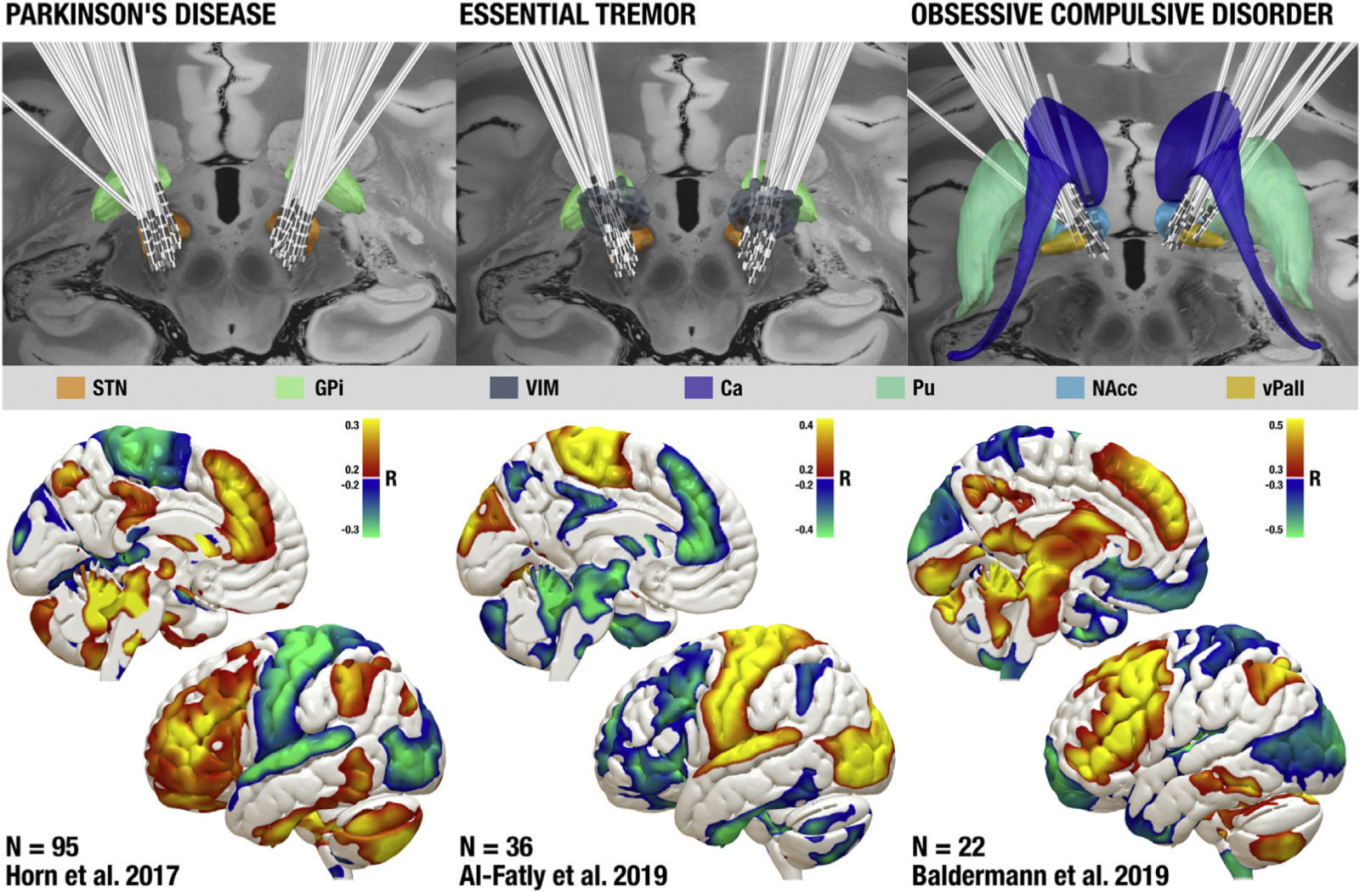
Functional connections with deep brain stimulation (DBS) sites that are correlated with clinical improvement. Top: DBS electrode locations targeting the subthalamic nucleus (STN) in patient’s with Parkinson’s disease (left), the ventral intermediate nucleus of the thalamus (VIM) in patients with essential tremor (middle), and the anterior limb of the internal capsule (ALIC) in patients with OCD (right). Bottom: brain regions whose functional connectivity to DBS sitesis correlated with clinical improvement. Positive correlations are shown in warm colors and negative correlations are shown in cool colors. DBS data are from previous studies (Al-Fatly et al., 2019; Baldermann et al., 2019b; Horn et al.,2017) and electrodes are displayed with axial slices from the 100um 7T postmortem MRI template (Edlow et al., 2019). Ca: Caudate nucleus, Pu: Putamen, NAcc: Nucleus Accumbens, vPall: ventral Pallidum.

Just as the normative connectome can lend insight into lesions causing a symptom, it can lend insight into DBS sites providing symptom relief. Identifying the “DBS site” is a bit more complicated than simply outlining a lesion, but tools are available to estimate the volume of tissue activated (VTA) by a DBS electrode set to specific electrical parameters (Dembek et al., 2017; Horn et al., 2019b; McIntyre et al., 2004). Once this DBS site is identified, one can use the normative connectcome nectome to identify the set of brain regions anatomically or functionally connected to the stimulation site. Connections that co-vary with clinical improvement can then be identified. This concept was first used to identify connections that co-varied with improvement in Parkinson’s symptoms following DBS to the STN (Horn et al.,2017). Since this time, the same approach has been used to further investigate DBS-induced improvements in Parkinson’s Disease (Horn et al., 2017; Joutsa et al., 2018a), as well as Dystonia (Corp et al., 2019; Okromelidze et al., 2020), Essential Tremor (Al-Fatly et al.,2019), epilepsy (Middlebrooks et al., 2018a)and OCD (Baldermann et al., 2019b; Li et al., 2020)(Fig. 3).

Unlike patients with incidental brain lesions, DBS surgeries are planned, providing an opportunity to collect individualized connectome data in each patient prior to neuromodulation. As such, several studies have now used individualized connectome data instead of normative connectome data to identify connections associated with DBS response (Akram et al., 2018;^2017^; Middlebrooks et al., 2018b, 2018c; Vanegas Arroyave et al., 2016). Table 1 gives a non-exhaustive overview about published connectomic DBS studies.

An important question is why we should bother using a connectome when seeking to understand and improve neuromodulation? Many studies havefoundclear relationships between DBS electrode locations and clinical improvement, without the need to add connectomic information. For instance, recent studies of STN-DBS for Parkinson’s Disease identified nearly the same optimal coordinate, including significant correlations between proximity to this coordinate and clinical improvement (Akram et al., 2017; Bot et al., 2018; Horn et al., 2019b; Nguyen et al., 2019). If such clear links between the *local* stimulation sites and clinical improvements exist, why should one bother with the human connectome? The remainder of this article focuses on answering this question.

## 3. Eight opportunities of connectomic neuromodulation

We highlight eight opportunities of combining neuromodulation with connectomics and append testable hypotheses to each opportunity. These eight opportunities include using the connectome data to understand the clinical effects of neuromodulation (#1, 2, 3, 5), individualize treatment (#4, 6 and 7), and advance our understanding of brain function (#8).

### 3.1. Opportunity #1: using connectomics to explain and predict clinical improvement

As mentioned above, studies have begun to investigate the relationship between clinical improvement following DBS and connectivity between the active stimulation site and the rest of the brain (Akram et al., 2018; 2017; Al-Fatly et al., 2019; Baldermann et al., 2019b; Calabrese et al., 2015b; Fernandes et al., 2015; Horn et al., 2017; Irmen et al., 2019; Joutsa et al., 2018a; Li et al., 2020; Vanegas Arroyave et al., 2016).

In doing so, we may understand which brain networks are responsible for mediating treatment response to neuromodulation. In turn, this could lend insight into both pathological features of underlying diseases and the therapeutic mechanism of action of DBS. In Parkinson’s Disease (PD), structural connectivity between the DBS site and the SMA and (negative) functional connectivity between the DBS site and primary motor cortex (M1) was associated with symptom improvement (Horn et al., 2017). In essential tremor, proximity of the DBS site to the dentatothalamic tract explained clinical improvement better than proximity to the traditional DBS target in the VIM (Calabrese et al., 2015b). In both diseases, connectivity profiles were correlated with clinical improvement in independent DBS patients or cohorts (Al-Fatly et al., 2019;Horn et al., 2017). In OCD, connectivity with the DBS site was able to explain about 30% of variance in clinical improvement in a split-half design (Baldermann et al., 2019b). A similar connectome-based approachhas been used to study clinical response to DBS in chronic pain (Fernandes et al., 2015), dystonia (Corp et al., 2019), depression (Choi et al., 2015; Riva-Posse et al.,2014), treatment-refractory epilepsy (Middlebrooks et al., 2018a) and Tourette’s Syndrome (Johnson et al., 2020). These studies established a direct–if correlational – link between clinical improvements and brain connectivity with the DBS site. We are now poised to test whether these connectivity profiles can predict clinical response in independent DBS cohorts in a prospective fashion, based solely on the location of the stimulation site and a map of the human connectome.

Testable hypothesis (#1): Connectivity between the neuromodulation site andother brain regions will prospectively predict improvement in clinical symptoms.

### 3.2. Opportunity #2: linking different DBS targets to the same network

**Fig. 4.**
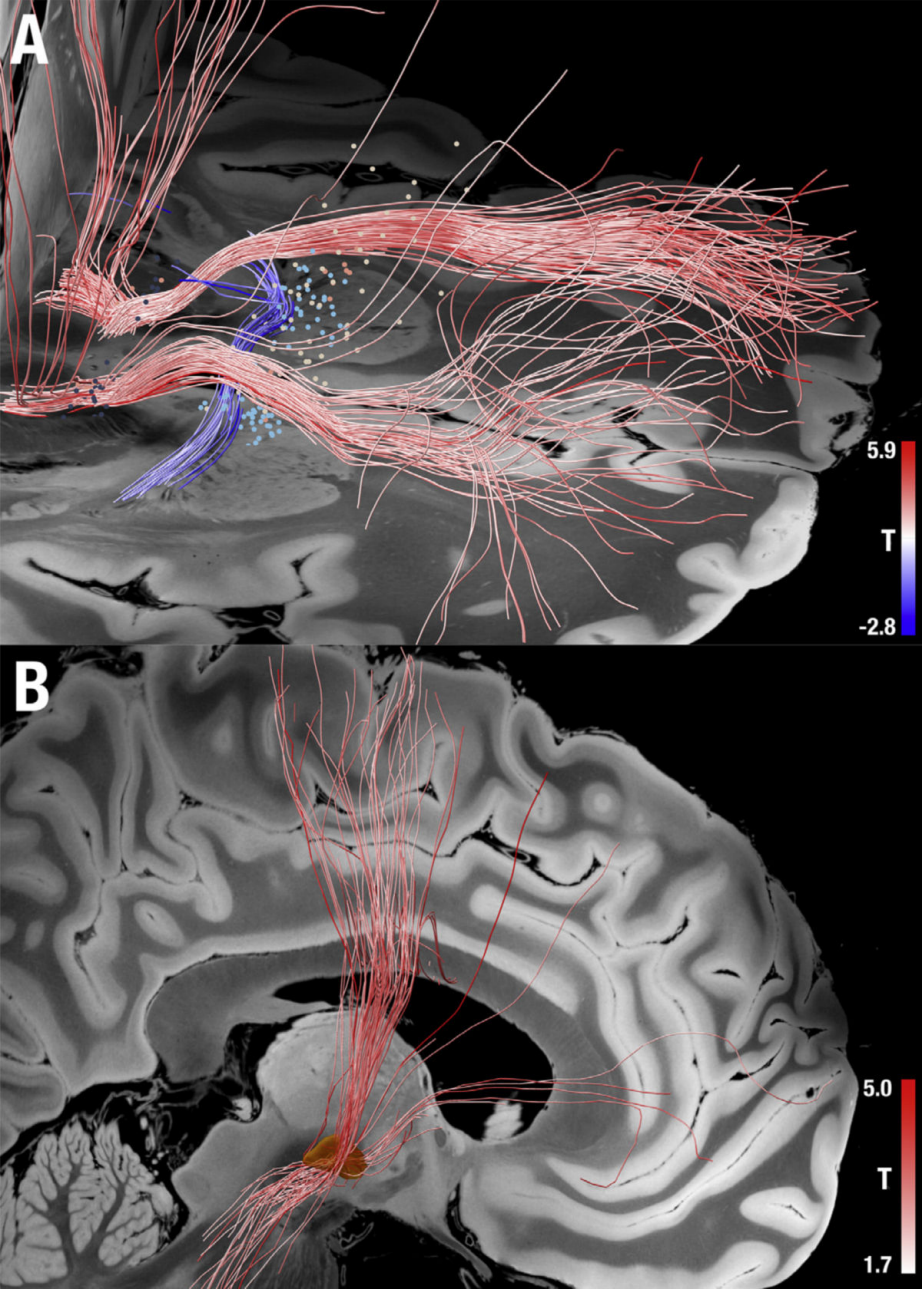
Filtering structural connectomes based on clinical improvement. (A) Active contact locations from 50 patients (four cohorts) that underwent DBS surgery for OCD to multiple different neuranatomical targets are shown as small spheres. The color of each sphere refers to the cohort (Li et al., 2020). Fiber tracts from a normative structural connectome were identified that traversed the stimulation site more frequently in patients with good clinical response (red) versus poor clinical response (blue). (B) The same method was applied to data from 51 patients that underwent STN-DBS for PD and identified the premotor hyperdirect pathway (red fibers) as being associated with better clinical response (Treu et al., 2020).

Brain connectivity measures can help link different brain stimulation sites that are used to treat the same disease or symptom to a common neuroanatomical substrate. For instance, Obsessive-Compulsive Disorder (OCD) has been treated with DBS using two different neuroanatomical targets: the anterior limb of the internal capsule (ALIC) (Baldermann et al.,2019b) and the STN (Li et al., 2020). Using the connectome, one can identify connections associated with clinical improvement at each target, and test whether the same connections underlie clinical improvement at both targets. Using a normative dMRI-based connectome, it was possible to identify common fiber tracts associated with clinical improvement across both DBS targets (Baldermannetal., 2019b; Li et al., 2020).In fact, tracts identified based on one target were associated with clinical improvement following DBS to the other target across multiple independent cohorts (Li et al., 2020)(Fig. 4).Similarly, there is evidence to suggest that different DBS targets for depression are part of a single anatomically connected circuit (Coenen et al., 2019b; Dougherty and Rauch, 2007; Dougherty et al., 2015). Thus, by investigating connections associated with clinical improvement across different DBS targets, one may identify a common network underlying therapeutic response.

It is worth noting that although connectivity may identify a common neuroanatomical substrate across different stimulation sites underlying a common clinical effect, this does not preclude the possibility of different effects at the different sites. A good example is a clinical trial carried out by Tyagi et al. in which both the STN and ALIC regions were targeted in the same OCD patients (4 implanted electrodes) (Tyagi et al.,2019). Stimulating either target led to similar reductions in obsessive-compulsive symptoms – suggesting a common circuit (Li et al., 2020). However, the STN target preferentially improved cognitive inflexibility while the ALIC target preferentially improved co-morbid depressive symptoms. This speaks for the notion that networks are symptom-specific (not disease-specific) and while two DBS sites may share one network and impact on certain symptoms (such as obsessive-compulsive symptoms), they may differ in their impacton other networks or symptoms.

Testable hypothesis (#2): Different DBS sites effective for the same symptom will be connected to a common brain network.

### 3.3. Opportunity #3: symptom specific networks

Brain disorders include a range of heterogenous symptoms that likely mapto different brain networks. Connectomic neuromodulation may help identify these networks and lead to symptom-specific treatments. In Parkinson’s Disease, Akram and colleagues showed that structural connections between the STNDBS site and supplementary motor area were associated with improvement in bradykinesia and rigidity, while structural connections to M1 were associated with improvement in tremor (Akram et al., 2017). Interestingly, connectivity to M1 was also associated with improved tremor following DBS to the ventrointer mediate nucleus (VIM) of the thalamus in patients with essential tremor (Akram et al., 2018; Al-Fatly et al., 2019). This suggests that we may need to stimulate different networks to treat different symptoms, paving the way for personalized therapy. For instance, tremor-dominant PD-patients could be treated with a slightly different STN DBS target than patients with predominant bradykinesia and rigidity. This same approach is being used to identify symptom-specific targets for transcranial magnetic stimulation for the treatment of depression (Siddiqi et al., 2020; Weigand et al., 2018). Dysphoric symptoms such as sadness respond best to TMS to one network, while anxiosomatic symptoms such as sleep and sexual interest respond best to TMS to a different network (Siddiqi et al., 2020; Weigand et al., 2018).

For patients with multiple symptoms that may require modulation of multiple different symptom-specific networks, more than one neuromodulation target may be needed. For example, one DBS trial used a parietal trajectory to target both the STN (for bradykinesia and rigidity) and the VIM/DRT (for tremor) (Reinacher et al., 2018). It is also possible to implant multiple electrodes targeting different networks and symptoms, such as GPi leads placed to control dyskinesias refractoryto STN DBS (Sriram et al.,2014).

Testable hypothesis (#3): Different connections with the neuromodulation site will be associated with improvement in different symptoms; different network targets will be needed to optimally improve different symptoms.

### 3.4. Opportunity #4: personalizing the connectome

In many of the aforementioned studies, network targets were identified using normative connectome data that was not derived from the individual patient (Al-Fatly et al., 2019; Baldermann etal., 2019b; Calabrese, 2016; Cash et al., 2019; Horn et al., 2017; Irmen et al., 2020; Petersen et al., 2019; Weigand et al., 2018). Normative connectomes have been derived from several different sources including ultra high-resolution postmortem MRI data (Aggarwal et al., 2013; Calabrese et al., 2015b), data from specialized MRI hardware optimized for connectome nectome imaging (Holmes et al., 2015; Setsompop et al., 2013; Van Essen et al., 2012; Yeo et al., 2011), and even tract atlases derived using augmented reality environments (Petersen et al., 2019) or from histological datasets (Alho et al., 2019). Normative connectomes are generally built from large datasets of up to 1000 individuals (Al-Fatly et al., 2019; Baldermann et al., 2019b; Holmes et al., 2015; Horn et al., 2017; Li et al., 2020; van Essen and Ugurbil, 2012; Weigand et al., 2018) and can be age- and disease-matched to patient cohorts of study (Ewert et al., 2018; Horn et al., 2017; Weigand et al., 2018). A combination of normative connectomes with tract atlases from other sources mentioned aboveis a promising way to account for limitations of either method (Li et al., 2020; Treu et al., 2020).

The benefit of using these normative connectome datasets is that they are generally higher in resolution and show better signal to noise than what can be acquired in individual patients using convention clinical MRI scanners. However, this approach ignores individual differences in connectivity that may be important in understanding neuromodulation effects (Akram et al., 2018; Fernandes et al., 2015; Lenglet et al., 2012; Petersen et al., 2017; van Hartevelt et al., 2014). An important opportunity is to move from normative connectome data to that from individual patients.

Many DBS studies have already used individualized connectivity data (Akram et al., 2018; 2017; Baldermann et al., 2019b; Fernandes et al., 2015; Kahan et al., 2014; Middlebrooks et al., 2018b; Tyagi et al., 2019; van Hartevelt et al., 2014). However, using individualized connectomes is challenging due to poor signal-to-noise and test-retest reliability. This was demonstrated nicely in a study by Petersen and colleagues that acquired dMRI data from the same subject ten times. In each scan, the authors used either the same or different fiber tracking algorithms to identify the peak in the STN most strongly structurally connected to motor-/premotor cortices (Petersen et al., 2017). Average distances between peaks identified on different days using the same approach were 0.5–1 mm and distances between peaks identified using different algorithms were 1.4mm. In a similar study, Jakab and colleagues scanned subjects on different MRI scanners and concluded that the test-retest variability (in surgically relevant bundles) caused by the MRI machine was similar or higher to the variability between subjects (Jakab et al., 2016).

Similar test-retest problems have been reported when using individualized functional connectivity to identify neuro modulation targets (Fox et al., 2013). Several methods have been introduced to improve the robustness of individualized functional connectomes (Fox et al.,2013; Kong et al., 2019; Wang et al., 2015), including simply collecting more functional connectivity data (Greene et al., 2019). One recent endeavor acquired 5 hours of rs-fMRI data per subject across 10 imaging sessions (Gordon et al.,2017). Acquiring such a vast amount of data for each patient undergoing DBS surgery faces obvious practical challenges. Still, a subsequent study used this dataset to learn more about DBS targets derived from individualized vs. normative connectome data (Greene et al., 2019).Namely, authors found that connectivity profiles of the VIM were consistent across individuals and related this to consistently high (>80%) DBS response rates in ET patients. In contrast, connectivity profiles of the GPi were more variable, which authors related to amore variable outcome of GPi DBS.

Recently, groups have begun to directly compare results using patient-specific vs. normative connectomes in DBS (Wang et al., 2020) or TMS (Cash et al., 2019). Both studies found no significant difference between the two connectomes in their ability to predict clinical outcomes, but noted a slight trend towards better prediction with individualized data.

Testable hypothesis (#4): Individualized connectomes will become more robust over time and will predict more variance in neuromodulation outcomes compared to normative connectomes.

### 3.5. Opportunity #5: mapping networks that lead to neuromodulation side-effects

Similar to mapping networks that lead to symptom improvements, it is possible to identify those that may lead to side-effects (second section of Table 1).On a local level, clinical experience has led to the heuristic that STN-DBS electrodes, if laterally placed, can lead to tetanic contractions and dysarthria, medially placed to paresthesia, ataxia, sweating and mydriasis, superiorly placed to freezing and akinesia and inferiorly placed to impulsivity and mania (Castrioto et al., 2013). Identifying connections and networks associated with such side-effects may helpus better understand their etiology and how they might be avoided. For instance, Al-Fatly et al. reported functional networks associated with the occurrence of ataxia and dysarthria in patients undergoing VIM-DBSfor ET (Al-Fatly et al., 2019). Here, occurrence of ataxia was associated with functional connectivity to a specific site in the vermis that had been previously associated with ataxia (Reich et al., 2016).

Irmen et al. demonstrated that in each of three PD cohorts undergoing STN-DBS at different centers, structural connectivity between DBS electrodes and the left prefrontal cortex was associated with the occurrence of depressive symptoms after surgery (Irmen et al., 2019). The finding was highly reproducible and allowed robust cross-predictions across cohorts. In OCD patients, weight change following DBS to the ALIC was associated with functional connectivity to the bed nucleusof the stria terminalis (Baldermann et al., 2019a). In a different report, stimulating electrodes that were connected to the periaqueductal grey and amygdala induced panic attacks (Elias et al., 2019). Suprathreshold stimulation of a patient suffering from anorexia nervosa with electrodes to the subcallosal cingulate led to a generalized seizure, which was attributed to connectivity between the stimulation site and bilateral hippocampi, cingulate gyri, and temporal lobes (Boutet et al., 2019a). Finally, a case-report of DBS to the centromedian nucleus for treatment of drug-resistant epilepsy reported occurrence of aggressiveness by a stimulation site that was connected to prefrontal cortex-bound white matter tracts (Yan et al., 2019).

These studies hint at a powerful future: choosing a stimulation site based on connectivity to therapeutic networks while avoiding stimulation sites connected to side-effect networks (Vorwerk et al., 2019). For instance, in PD, a tremor-dominant patient could be optimally treated when stimulated at a coordinate that is maximally connected to a “tremor-network” but not connected to a network associated with side-effects such as depressive symptoms.

Testable hypothesis(#5): Different connections with the neuromodulation site will be associated with different neuromodulation side effects; avoiding these connections will help avoid side effects.

### 3.6. Opportunity #6: connectomics guiding DBS programming and neurosurgery

Once optimal connectivity profiles are established, these connectivity profiles might be used to guide DBS programming. Such work could lead to algorithms that automatically find optimal DBS programming parameters by maximizing impact on target networks while minimizing impact on side effect networks. For instance, one study evaluated an algorithm that would automatically find parameters that increased impact on the VIM while avoiding the internal capsule and ventralis caudalis dalis nucleus of the thalamus (Vorwerk et al., 2019). While the capsule is a white-matter tract, the study still focused on local features rather than exploiting the connectome concept. First feasibility studies that automatically estimated DBS settings by maximizing connectivity overlap with a personalized set of network targets have been performed (Krishna et al., 2019).

Just as connectivity might be used to guide DBS programming, it could be used to guide DBS surgery. For example, some surgeons have already begun to use individualized dMRI data to target the dentatothalamic tract instead of the VIM in ET (Coenen et al., 2011; 2016) or target it in addition to the STN in PD patients with tremor (Coenen et al., 2016; Reinacher et al., 2018). In depression, surgeons have used individualized dMRI data to target the medial forebrain bundle (Coenen et al., 2009; Schlaepfer et al., 2013) or the intersection of forceps minor, cingulum bundle and uncinate fasciculus (Noecker et al., 2018; Choi et al., 2015; Riva-Posse et al., 2017; 2014), the latter of which has led to improved open-label response rates(Riva-Posse et al., 2017). To the best of our knowledge, rs-fMRI has not yet been used to inform individual DBS targets in clinical practice – although the concept has been explored (Al-Fatly et al., 2019; Anderson et al., 2011; Greene et al., 2019) and used to guide TMS (Cole et al., 2020).

Similar to the above, connectivity may be used to guide neurosurgical ablations or therapeutic lesions. Although DBS has largely replaced lesions for many indications, new technologies that allow one to create lesions without surgical incision are gaining popularity. In particular, MR-guided focused ultrasound (MRgFUS) uses acoustic soundwaves to create focal brain lesions. This method was first introduced in patients suffering from chronic neuropathic pain (Martin et al., 2009), has been FDA approved for treatment of essential tremor (Elias et al., 2016) and tremor-predominant Parkinson’s disease (Bond et al., 2017), and is being explored as a treatment for other disorders including psychiatric conditions. Since MRgFUS lesioning is guided by imaging rather than electrophysiology, it could benefit greatly from integration with imaging resources such as the human connectome. The normative structural connectome has already been used to investigate connections associated with side-effects following MRgFUS lesioning of the thalamus for Essential Tremor (Boutet et al., 2018) and connections associated with clinical benefit following MRgFUS lesioning of the anterior limb of the internal capsule for OCD or Major Depressive Disorder (Davidson et al., 2020).

It is worth noting that there should be a higher bar for using connectivity to guide DBS surgery or MRgFUS versus DBS programming (Coenen et al., 2019b). Once a nelectrode has been implanted, its location cannot be easily changed, and lesions from MRgFUS are irrversible. In contrast,DBS programming can be easily adjusted if a connectome-based hypothesis turns out to be wrong. The risk versus benefit of incorporating connectivity information into surgical planning should be carefully weighed by experienced physicians, with ongoing studies to determine the value of this information.

Testable hypothesis (#6): Connectome-based DBS programming will allow for faster optimization of DBS parameters and fewer side effects; connectome-based neurosurgery will inform new targets and surgical trajectories that improve clinical outcomes.

### 3.7. Opportunity #7: bridging invasive and noninvasive brain stimulation

Most of the aforementioned studies applied invasive (DBS) while others have applied noninvasive (TMS) strategies to modulate brain activity. In 2014, Fox and colleagues demonstrated that across 14 diseases, invasive and noninvasive neuromodulation sites used to treat the same symptoms are part of the same connected brain network (Fox et al., 2014).This suggests that one might modulate the same network using either TMS (cortically) or DBS (subcortically) to improve the same symptom. For example, the most popular DBS target in PD (the STN) was functionally connected to the SMA and primary motor cortex, two TMS sites with beneficial effects on PD symptoms. Later, it was found that DBS sites that are the most connected to SMA and primary motor cortex resulted in better clinical improvement (Horn et al., 2017). Similarly, the most popular DBS target for depression (Broadman’s area 25 / subcallosal cingulate cortex) was functionally connected to the DLPFC, a TMS site with beneficial effects on depression (Fox et al., 2014; 2012). Later, it was found that TMS sites that are the most connected to BA25/SCC resulted in better clinical improvement (Cash et al., 2019;Weigand et al., 2018). Whether this concept holds true for other conditions reported in this 2014 paper such as addiction, Alzheimer’s disease, anorexia, dystonia, epilepsy, OCD, pain or Tourette’s Disease remains to be formally investigated.

Testable hypothesis (#7): Neuromodulation sites effective for the same symptom will be part of the same connected brain network across different neuromodulation modalities.

### 3.8. Opportunity #8: a window to understand the brain

So far, our review has focused on clinical applications of how connectomics could improve neuromodulation treatment. However, the same concepts can be applied to advance systems and cognitive neuroscience. For example, it has recently become possible to acquire fMRI scans in patients while their DBS system is switched on. This allows for studies of remote *changes* on other brain regions, and on the functional connectome induced by DBS (Boutet et al., 2019b;Horn et al., 2019c; Jech et al., 2001; Kahan et al., 2014; Mueller et al., 2013). For example, STN-DBS appears to increase connectivity between the sensorimotor cortex and thalamus and decrease connectivity between the striatum and cerebellum (Horn et al., 2019c; Kahan et al.,2014). These studies demonstrate how DBS could be used to modulate neural activity in awake humans and study the consequences to better understand the brain in general. This concept has been used to explore novel DBS targets and to gain better understanding of physiology and pathology (Saenger et al.,2017).

A second line of research again applied normative connectomes with DBS –but this time to address questions of cognitive neuroscience(third section in Table 1). For instance, Neumann et al. showed that specific connections of the DBS electrodes in the STN would lead to changes in movement velocity vs. reaction times in a motor task (Neumann et al., 2018). Similarly, functional connectivity between STN-DBS electrodes and a specific site in the ipsilateral cerebellum was associated with restoring motor learning in PD patients (de Almeida Marcelino et al., 2019).These studies show utility of the connectomic neuromodulation concept above and beyond addressing clinical questions.

Testable hypothesis (#8): Changes of the functional connectome under neuromodulation will lead to a better understanding of brain function.

## 4. Limitations of connectomic neuromodulation

Although connectomic neuromodulation bears many promising opportunities as outlined above, there are important limitations. First, MRI-based connectivity techniques (dMRI and rs-fcMRI) are not sensitive to the directionality of connectivity (e.g. inputs versus outputs), specific neuronal subtypes, local micro-circuits, or different neurotransmitters. As such, the same neuromodulation stimulus applied to different ent brain regions could lead to different results even if their MRI-based connectivity profiles were exactly the same. Second, all connectomic neuromodulation studies published to date are based on correlation, i.e. connectivity profiles with the stimulation site are identified that *correlate* with clinical outcome. Whether the identified connections, or modulation of connected brain regions, are causally linked to therapeutic outcome is uncertain. Similarly,it is hard to differentiate whether clinical effects are due to connectivity with the neuromodulation site or local effects of the neuromodulation site, as the two are intrinsically linked. For instance, moving a DBS electrode more anteromedial in the subthalamic nucleus will lead to a (nonlinear) shift of connectivity to more frontal regions. Segregating whether clinical results of such a shift result from modulating different functional zones of the STN or different networks connected to different STN subregions is difficult. Combining information from multiple different brain stimulation sites that are part of the same network may help resolve this ambiguity (Fox et al., 2014; Li et al., 2020).

There are several limitations of dMRI, which is based on water different fusion and only approximates white matter or anatomical connectivity. As such, it is not perfectly suited to measure connectivity strength between two areas. Stream line counts and average fractional anisotropy values along connecting tracts have been used to estimate the degree of structural connectivity but both measures can be unreliable. On average every valid connection present in a typical single-subject diffusion MRI based tractogram is matched by four invalid (false positive) connections (Maier-Hein et al., 2017). Thus, when using tractography to identify “novel” connections (Hosp et al., 2019; Milardi et al., 2019; Quartarone et al., 2019), the chance of getting wrong answers is higher than of getting true answers (Maier-Hein et al., 2017; Petersen et al., 2019). Moreover, myelinated long tracts are overrepresented and very short and thin bundles can be hardly reconstructed, if at all (Edlow et al., 2019; Horn et al., 2019a; Petersen et al., 2019). However, these latter bundles (such as the ansa lenticularis, the lenticular fascicle, Edinger’s comb system or Wilson’s pencils in the striatum) may play a crucial role in mediating DBS effects (Horn et al., 2019a).

There are also many limitations of rs-fcMRI, which is based on slow fluctuations in blood flow and oxygenation. These fluctuations are only an indirect reflection of underlying neural activity, and can be contaminated by many non-neuronal sources of noise (Fox and Raichle, 2007b; Murphy and Fox, 2017). Rs-fcMRI is also insensitive to brain oscillations occurring on faster time scales (Buzsáki, 2006), including beta oscillations that may play an important role in brain disorders such as PD (Kühn et al., 2006).

Normative connectomes share all limitations of diffusion-/functional MRI but come with an additional limitation in that they do not account for individual differences in brain connectivity. Individualized connectomes come with significant limitations in signal to noise and reproducibility. As such, connectomic DBS studies have explained a maximum of 30–40% of variance in clinical improvement across independent datasets (e.g. *R* = 0.55–0.69 in (Baldermann et al., 2019b), also see Table 1).This variance compares favorably with other predictors of DBS outcomes in independent datasets, including L-dopa response (Horn et al., 2017; Irmen et al., 2020), but may still fall short of clinical utility. Reasons why explained variance is not higher include limitations of the connectome, nectome, but also limitations of our clinical outcome measures and the fact that clinical outcomes are dependent on many factors besides the neuromodulation site including disease-subtype, specific symptoms, comorbidities, age, etc. For example, one PD patient in which we predicted a good DBS outcome based on their stimulation site and connectivity did much worse than expected in the setting of severe depression. Once the patient’s depression improved, their motor scores also improvedto match the connectome-based prediction (Horn et al., 2017).

## 5. Conclusions

Connectomic neuromodulation provides numerous opportunities to better understand and predict clinical outcomes, to personalize neuromodulation therapy, and to integrate findings across neuromodulation targets and modalities. Each opportunity allows for testable hypotheses towards improving neuromodulation treatment. However, there remain important limitations, and caution is warranted as novel imaging methods are incorporated into clinical practice – especially in the operating room. We see great potential in connectomic neuromodulation, andwe look forward to ongoing research and clinical trials designed to test the value of this approach.

## Figures and Tables

**Table 1:** Overview of published connectomic DBS studies. The table separates studies that focused on clinical efficacy, side effects, or behavioral changes. For studies that predicted changes across patients (i.e. in out-of-sample data), the amount of variance explained is reported. For abbreviations, see index above.

**Disease**	**Study**	**Outcome variable**	**Target**	***N***	**Connectivity type**	**Connectivity data**	**Main processing tools**	**% variance explained (out-of-sample data)**

PD	(Vanegas Arroyave et al., 2016)	UPDRS-III	STN	22	Structural	Individualized	DBSproc, TORTOISE, FATCAT	*N/A*
	(Akram et al., 2017)	Bradykinesia/Rigidity/Tremor	STN	20	Structural	Individualized	SureTune and FSL	*N/A*
	(Horn et al., 2017)	UPDRS-III	STN	95	Structural and Functional	Normative/ *	Lead-DBS	~26 %
	(Middlebrooks et al., 2018c)	UPDRS-III	GPi	11	Structural	Individualized	Lead-DBS and FSL	*N/A*
	(Avecillas-Chasin, 2019)	UPDRS-III Subscores	STN	43	Structural	Normative *	Lead-DBS and MRTrix	*N/A*
ET/PD	(Akram et al., 2018)	TRS	VIM	9	Structural	Individualized	SureTune and FSL	*N/A*
ET	(Coenen et al., 2011) (Calabrese et al., 2015a)	TRS Custom Scale	DRT VIM	1	Structural Structural	Individualized Normative $	StealthViz DTI FSL	*N/A* ~11 %
	(Middlebrooks et al., 2018b)	TRS	VIM	40	Structural	Individualized	Lead-DBS and FSL	*N/A*
	(Al-Fatly et al., 2019)	TRS	VIM	33	Structural	Normative	Lead-DBS	~13–16 %
Voice Tremor	(Avecillas Chasin et al., 2019)	VT score	VIM	7	Structural	Normative	Lead-DBS and MRTrix	*N/A*
Dystonia	(Okromelidze et al., 2020)	UDRS	GPi	39	Structural and Functional	Normative	Lead-DBS	*N/A*
OCD	(Hartmann et al., 2016)	Y-BOCS	ALIC/NAcc	6	Structural	Normative †	SCIRun, Comsol, FSL	*N/A*
	(Coenen et al., 2017)	Y-BOCS	slMFB	2	Structural	Individualized	StealthViz DTI	*N/A*
	(Baldermann et al., 2019b)	Y-BOCS	ALIC	22	Structural	Individualized and Normative	Lead-DBS and DSI Studio	~30–47 %
	(Liebrand et al., 2019)	Y-BOCS	ALIC	12	Structural	Individualized	FSL	~34%
	(Li et al., 2020)	Y-BOCS	ALIC, STN, NAcc	50	Structural	Normative	Lead-DBS	~25–56 %
TS	(Brito et al., 2019)	YGTSS	CM-Pf	5	Structural	Normative	Lead-DBS	*N/A*
	(Johnson et al., 2020)	YGTSS	CM, GPi	67	Structural	Normative	SCIRun and FSL	*~14%*
Depression	(Schlaepfer et al., 2013)	MADRS	slMFB	7	Structural	Individualized	StealthViz DTI	*N/A*
	(Riva-Posse et al., 2014)	HDRS-17	Cg25	17	Structural	Individualized	FSL	*N/A*
	(Choi et al., 2015)	Self-reports	Cg25	9	Structural	Individualized	FSL	*N/A*
	(Bewernick et al., 2017)	MADRS	slMFB	8	Structural	Individualized	StealthViz DTI	*N/A*
	(Riva-Posse et al., 2017)	HDRS-17	Cg25	11	Structural	Individualized	StimVision	*N/A*
	(Coenen et al., 2019a)	MADRS	slMFB	24	Structural	Individualized and Normative	CAT12, Gibbstracker	*N/A*
Epilepsy	(Middlebrooks et al., 2018a)	Seizure frequency	ANT	6	Functional	Normative	Lead-DBS	*N/A*
Pain	(Fernandes et al., 2015)	Successful/Unsuccessful	Cingulate	6	Structural	Individualized	FSL	*N/A*

**Disease**	**Study**	**Side Effect**	**Target**	***N***	**Connectivity type**	**Connectivity data**	**Main processing tools**	**% variance explained (out-of-sample data)**

PD	(Irmen et al., 2020)	Depression (BDI)	STN	116	Structural	Normative	Lead-DBS	~10–33%
	(Cury et al., 2020)	Pain	STN	32	Structural	Normative	Lead-DBS	N/A
PD	(Mosley et al. 2020)	Impulsivity	STN	55	Structural	Individualized and Normative	Lead-DBS, MRtrix3	N/A
ET	(Al-Fatly et al., 2019)	Ataxia and Dysarthria	VIM	33	Structural	Normative	Lead-DBS	*N/A*
OCD	(Baldermann et al., 2019a)	Body Weight	ALIC	25	Functional	Normative	Lead-DBS	*N/A*
	(Elias et al., 2019)	Panic	ITP	1	Functional	Normative	Lead-DBS	*N/A*
Depression	(Boutet et al., 2019a)	Seizures	Cg25	1	Functional	Normative	Lead-DBS	*N/A*
Epilepsy	(Yan et al., 2019)	Aggressiveness	CM	1	Structural	Normative	Lead-DBS	*N/A*

**Disease**	**Study**	**Behavioral Effect**	**Target**	***N***	**Connectivity type**	**Connectivity data**	**Main processing tools**	**% variance explained (out-of-sample data)**

PD	(Neumann et al., 2018)	Reaction time and Movement velocity	STN	20	Structural	Normative	Lead-DBS	53–76%
	(de Almeida Marcelino et al., 2019)	Motor Learning	STN	20	Functional	Normative	Lead-DBS	33 %

* = Age and disease matched group connectome used.

$ = a postmortem connectome was used.

† = a single (unrelated) diffusion scan was used for all patients.

## Abbreviations

ALIC: Anterior limb of the internal capsule
ANT: Anterior nucleus of the thalamus
CM-Pf: Centromedian nucleus and Parafascicular nucleus of the thalamus
DBS: Deep Brain Stimulation
DR(T)T: Dentatorubrothalamic tract
ET: Essential Tremor
FATCAT: Functional And Tractographic Connectivity Analysis Toolbox
FSL: FMRIB Software library
GPi/GPe: internal/external pallidum
ITP: inferior thalamic peduncle
M1: Primary motor cortex
MADRS: Montgomery– Åsberg Depression Rating Scale
MNI: Montreal Neurological Institute
MRI: Magnetic Resonance Imaging
NAcc: Nucleus Accumbens
OCD: Obsessive Compulsive Disorder
PET: Positron Emmision Tomography
PD: Parkinson’s Disease
PFC: Prefrontal córtex
PLI: Polarized Light Imaging
slMFB: superolateral branch ofthe medial forebrain bundle (as defined by Coenen et al. 2009)
SN: Substantia Nigra
STN: Subthalamic Nucleus
SMA: Supplementary Motor Area
TORTOISE: Tolerably Obsessive Registration and Tensor Optimization Indolent Software Ensemble
UPDRS: Unified Parkinson’s Disease Rating Scale (part III refers to motor assessment)
VIM: Ventral Intermediate Nucleus
VTA: Volume of Tissue Activated
Y-BOCS: Yale–Brown Obsessive Compulsive Scale
YGTSS: Yale Global Tic Severity Scale
